# Myocardial Infarction after Kidney Transplantation: A Risk and Specific Profile Analysis from a Nationwide French Medical Information Database

**DOI:** 10.3390/jcm9103356

**Published:** 2020-10-19

**Authors:** Romain Didier, Hermann Yao, Mathieu Legendre, Jean Michel Halimi, Jean Michel Rebibou, Julien Herbert, Marianne Zeller, Laurent Fauchier, Yves Cottin

**Affiliations:** 1 Department of Cardiology, University Teaching Hospital Burgundy, 21000 Dijon, France; romain.didier@chu-dijon.fr (R.D.); hermannyao@gmail.com (H.Y.); 2 Department of Nephrology, University Teaching Hospital Burgundy, 21000 Dijon, France; mathieu.legendre@chu-dijon.fr (M.L.); jean-michel.rebibou@chu-dijon.fr (J.M.R.); 3 Department of Nephrology, University Teaching Hospital of Trousseau and University François Rabelais University, 37000 Tours, France; jean-michel.halimi@univ-tours.fr; 4 Department of Cardiology, University Teaching Hospital of Trousseau and EA7505, University François Rabelais University, 37000 Tours, France; j.herbert@chu-tours.fr (J.H.); laurent.fauchier@univ-tours.fr (L.F.); 5 Department of Informatics and Epidemiology, University Teaching Hospital of Trousseau and EA7505, University François Rabelais University, 37000 Tours, France; 6 PEC2 Research Team, EA 7460, Department of Health Sciences, University of Burgundy Franche Comté, 25000 Besançon, France; marianne.zeller@chu-dijon.fr

**Keywords:** myocardial infarction, renal transplant recipients, all-cause of mortality, follow ow-up, cardiovascular death

## Abstract

Introduction: Renal transplant recipients have a high peri-operative risk for cardiovascular events. The post-transplantation period also carries a risk of myocardial infarction (MI). Coronary artery disease (CAD) is a leading cause of death in these patients. We aimed to assess the risk of MI, the specific morbidity profile of MI after transplantation as well as the long-term prognosis after MI in renal transplantation (RT) patients regarding cardiovascular (CV) death and all-cause death. Methods: From a French national medical information database, all of the patients seen in French hospitals in 2013 with at least 5-years follow-up were retrospectively identified and patients without transplantation but with previous dialysis at baseline were excluded. There were 17,526 patients with RT and 3,288,857 with no RT. Results: Among these patients, 1020 in the RT group (5.8%), and 93,320 in the non-RT group (2.8%) suffered acute MI during a median follow-up of 5.4 years. After multivariable adjustment, risk of MI was higher in RT patients than in non-RT patients (HR 1.45, IC 95% 1.35–1.55). The mean age was 59.5 years for transplant patients with MI, and 70.6 years for the reference population with MI (*p* < 0.0001). MI patients with RT (vs. non RT patients) were more likely to have hypertension, diabetes dyslipidemia, and peripheral artery disease (76.0% vs. 48.1%, 38.7% vs. 25.2%, 33.2% vs. 23.2%, and 31.2% vs. 17.3%, respectively, *p* < 0.0001). Incidence of non ST-elevation MI (NSTEMI) was higher in RT patients while incidence of ST-elevation MI (STEMI) was higher in patients without RT. In unadjusted analysis, risk of all-cause death and CV death within the first month after MI were higher in patients without RT (18% vs. 11.1% *p* < 0.0001 and 12.3% vs. 7.8%, *p* < 0.0001, respectively). However, multivariable analysis indicated that risk of all-cause death was higher in patients with RT than in those with no RT (adjusted HR 1.15 IC 95% 1.03–1.28). Conclusions: MI is not an uncommon complication after RT (incidence of around 5.8% after 5 years). RT is independently associated with a 45% higher risk of MI than in patients without RT, with a predominance of NSTEMI. MI in patients with RT is independently associated with a 15% higher risk of all-cause death than that in patients with MI and no RT.

## 1. Introduction

In renal transplant recipients, coronary artery disease (CAD) is still a leading cause of death and the second leading cause of transplant loss [1,2,3,4,5,6]. The incidence of CAD among renal transplant (RT) patients is higher than in the general population, ranging from 5–8%, with a cumulative incidence of 1.5% per year [7]. A recent study highlighted that one significantly calcified lesion was present in 75.7% of patients with RT vs. 15.8% of control patients on patients referred for coronary angiography [8].

The post-transplantation period carries a high risk of myocardial infarction (MI), particularly during the first year [7,9]. Patients from the Medicare registry have a MI incidence of 2.27% during the first year after renal transplantation [7]. Gill et al. reported, in a cohort of 600 patients, a significant increase in the incidence of cardiovascular (CV) events within the first year post-transplantation (39.6/100 patient-years, 95% CI 20.6–76.1) compared with the pre-transplantation period [9]. More than 10% of patients with RT experienced an acute MI within three years of transplantation, as reported by Lentine et al. [10]. Recent trends reported that among the 210,327 RT patients, 3.2% died within one year, mainly with CV death (24.7%) [4]. In addition, a study carried out among 780 RT patients showed a 3.5% incidence of MI with 50% mortality rate, and an increased risk of MI in the first three months post transplantation [11]. The international multicenter PORT study (Patient Outcomes in Renal Transplantation), published in 2010, reported specific risk factors for MI after RT in 23,575 patients: acute rejection, pre-transplantation duration of dialysis, and diabetes [12].

Most studies have compared kidney transplantation with patients on dialysis awaiting a transplant, which is already a high-risk population [8,13].

Based on a nationwide database, among patients hospitalized for any reason in 2013, we aimed to assess the risk of MI and the specific profile of MI after kidney transplantation as well as the long-term prognosis after MI regarding cardiovascular death and all-cause death during follow-up in this high-risk sub-population.

## 2. Patients and Methods

### 2.1. Study Design

This retrospective cohort study was based on the French administrative hospital discharge database (Programme de Médicalisation des Systèmes d’Information; PMSI), which collects hospital care data for the entire French population.

This study was conducted retrospectively, so there was no impact on patient care. Approval from a local ethics committee was not required because all data were anonymized. The French Data Protection Authority granted access to the PMSI data, and procedures for data collection and management were approved by the independent national ethics committee for human rights in France (Commission Nationale de l’Informatique et des Libertés; CNIL), which ensures that all information is kept confidential and anonymous in compliance with the Declaration of Helsinki (authorization number No. 1897139).

This database, which is used for hospital funding, records medical information for all patients who attend any of the 1546 French healthcare facilities, whether public or private.

### 2.2. Selection Criteria and Follow-Up

Among the 11,719,809 patients admitted in French hospitals in 2013, we selected patients aged over 18 years old with at least five years of subsequent follow-up (or with an event of interest in the interim) (n = 3,381,472). We excluded patients with dialysis and no kidney transplant at baseline (n = 75,089). Patients were all included in 2013 and then followed-up for at least five years until 30 June 2019 for the occurrence of outcomes. The endpoints (myocardial infarction, all-cause death, and cardiovascular death) were evaluated with follow-up starting from the date of first hospitalization in 2013 until the date of each specific outcome or date of last news in the absence of the outcome. Information on outcomes during follow-up was obtained by analyzing the PMSI codes for each patient. All-cause death and myocardial infarction were identified using their respective ICD-10 codes (international classification of diseases). Mode of death (cardiovascular or non-cardiovascular) was identified based on the main diagnosis during hospitalization, resulting in death based on ICD-10 codes (for cardiovascular death: I00–I99–Diseases of the heart and circulatory system).

### 2.3. Data Collection and Outcomes

All patients admitted in France in 2013 were collected from the PMSI using the annually updated versions of the ICD10. RT patients were identified using the code Z940, and Z992 + JVJ004 for dialysis.

**Figure 1:**
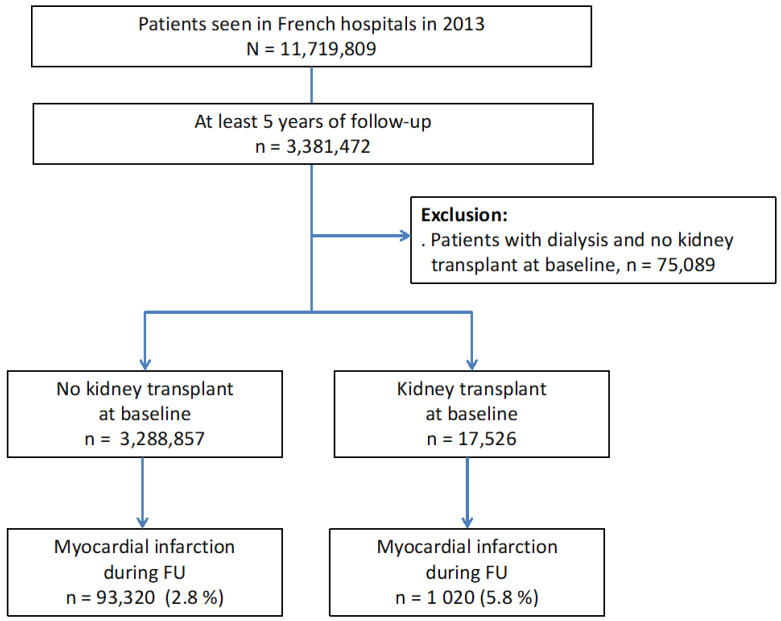
Flow chart of the study describing the incidence of MI in patients with kidney transplant or no kidney transplant seen in French hospitals in 2013 with at least five years of follow-up. (FU = Follow up).

Among the 3,306,383 patients, we considered two groups according to whether they had renal transplant at baseline (n = 17,526, RT) or not (n = 3,288,857, no RT) (Figure 1). This “Baseline population” was compared on demographic variables including age and sex. We also collected cardiovascular risk factors and comorbidities, history of CAD, history of MI, previous percutaneous coronary intervention (PCI), or coronary artery bypass graft (CABG) surgery. Regarding outcomes of interest, we analyzed incidence of myocardial infarction, all-cause death, and cardiovascular death. MI during follow-up was identified using the code I21 and a distinction was made between the two types of MI STEMI (ST-elevation MI) and NSTEMI (non ST-elevation MI).

A total 94,340 of these patients were then analyzed because of MI during follow up (Figure 1). In this “MI population”, we aimed to compare the same demographic variables and we analyzed time from kidney transplantation to the onset of MI. The all-cause and cardiovascular death over 0–8 and 0–30 days post MI, and during longer term follow-up were also studied.

### 2.4. Statistical Analysis

Incidence of outcomes was analyzed by a proportional hazard regression model to calculate hazard ratio (HR) with 95% confidence intervals (CI). Multivariate adjustment was performed using a Cox analysis on all characteristics of Table 1. We also used a model by Fine and Gray for analyzing competing risks for (1) myocardial infarction and all-cause death and (2) cardiovascular and non-cardiovascular death. All comparisons with *p* < 0.05 were considered statistically significant. Analyses were performed using Enterprise Guide 7.1 (SAS Institute Inc., SAS Campus Drive, Cary, NC, USA) and STATA version 12.0 (Stata Corp, College Station, TX, USA).

## 3. Results

### 3.1. Baseline Population

Over the study period, 3,306,383 patients were included in the study. Among them, 17,526 were renal transplant recipients (Figure 1). Time from transplantation to inclusion in the study in the 17,526 patients with RT at the baseline was 25.7 ± 12.9 months (median 30.0, interquartile 16.4–35.9 months). Table 1 summarizes the characteristics of the patients with RT or no RT at baseline.

Male gender was predominant in RT patients (60.4% vs. 46.5%, *p* < 0.0001). Patients without RT were significantly older than RT patients (59.1 yo vs. 55.4 yo, *p* < 0.0001). RT patients were more likely to have hypertension (70.4% vs. 29.5%, *p* < 0.0001), diabetes (28.3% vs. 13.2% *p* < 0.0001), dyslipidemia (25.1% vs. 12.7% *p* < 0.0001), obesity (12.3% vs. 10.3%, *p* < 0.0001), and AF was significantly higher in RT patients (11.4% vs. 9.4%, *p* < 0.0001). History of cardiovascular disease (CAD, previous MI, previous PCI and CABG surgery) were significantly more frequent in RT patients (*p* < 0.0001 respectively) (Table 1). Patients with RT were more likely to have a history of heart failure (22.2% vs. 9.9%, *p* < 0.0001), history of pulmonary edema (3.9 vs. 0.7%, *p* < 0.0001), and valvular heart disease (6.0% vs. 3.5%, *p* < 0.0001). (Table 1).

During follow-up, cardiovascular deaths occurred significantly more frequently in RT patients (6.0% vs. 5.6%, *p* = 0.01), while all causes-deaths were more frequently reported in patients without RT (27.7% vs. 25.7%, *p* < 0.0001) (Table 1).

**Figure 2:**
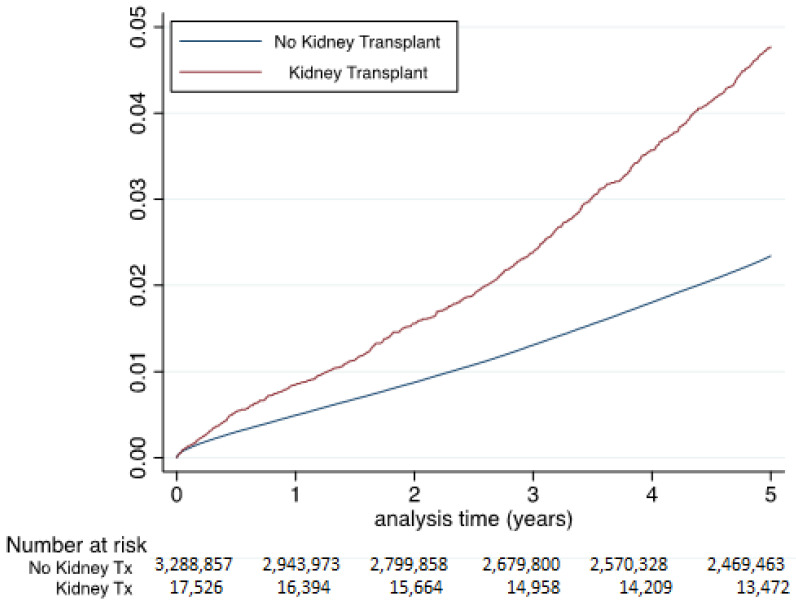
Cumulative incidence of myocardial infarction (MI) in patients seen in French hospitals in 2013 with at least five years of follow-up included in the study according to renal transplant (94,340 MI during follow-up of 4.6 ± 1.8 years, median 5.4, IQR 5.0–5.8 years). Hazard ratio 1.69, 95% CI 1.58–1.79 for renal transplant vs. no renal transplant. Adjusted hazard ratio 1.45, 95% CI 1.35–1.55, (adjustment on all characteristics of Table 1). Ordinate Axis = Incidence of myocardial infarction. (Kidney Tx = Kidney transplant; IQR = Interquartile Range).

Cumulative incidence of MI was significantly higher in RT patients (unadjusted HR 1.69, 95% CI 1.58–1.79). Multivariable adjustment was consistent with the univariate analysis (adjusted HR 1.45, 95% CI 1.35–1.55) (Figure 2). Fine and Gray model for competing risks of MI and all-cause provided similar findings (hazard ratio 1.86, 95% CI 1.74–1.97).

### 3.2. Myocardial infarct Population

During the follow-up period, among the 3,306,383 patients in the baseline population, 94,340 had a MI (1020 RT patients, 93,320 non-RT patients), representing 5.8% of RT patients and 2.8% of non-RT patients (Figure 1).

MI patients with RT were younger than those without RT (59.5 yo vs. 70.6 yo, *p* < 0.0001), with a male predominance (69.4% vs. 62.8%, *p* < 0.0001). Hypertension (76.0% vs. 48.1%, *p* < 0.0001), diabetes (38.7% vs. 25.2%, *p* < 0.0001), and dyslipidemia (33.2% vs. 23.2%, *p*< 0.0001) were more common in RT patients (Table 2).

**Figure 3:**
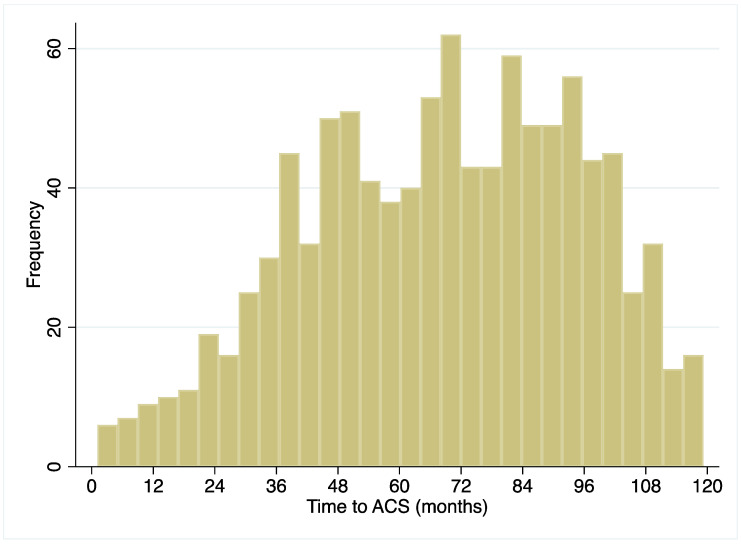
Time to the onset of the MI from renal transplant in 1020 patients with renal transplant and MI during follow-up. Mean 68 ± 27 months; median 70, IQR 48–90 months. (ACS = Acute coronary syndrome).

STEMI occurred more frequently in non-RT patients (49.1% vs. 43.9% *p* = 0.001), while a higher rate of NSTEMI was reported in RT patients (56.1 vs. 50.9%, *p* = 0.001). Occurrence of heart failure in the acute phase of MI was more frequent in the non-RT patients (23.8% vs. 17.6%, *p* < 0.0001). (Table 2). The time from kidney transplant to the onset of the myocardial infarction was 68 ± 27 months (median 70, IQR 48–90 months) (Figure 3).

**Figure 4:**
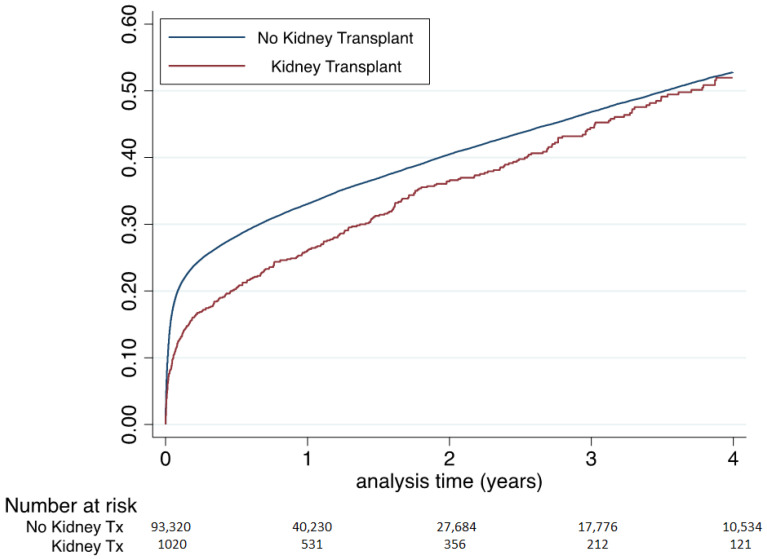
Cumulative incidence of all-cause death in patients seen in French hospitals in 2013 with myocardial infarction during follow-up according to renal transplant (36,026 deaths during follow-up of 1.4 ± 1.7 years, median 0.6, IQR 0.1–2.4 years). Hazard ratio 0.86, 95% CI 0.77–0.95 for renal transplant vs. no renal transplant. Adjusted hazard ratio 1.15, 95% CI 1.03–1.28 (adjustment on all characteristics of Table 1). Ordinate Axis = Incidence of all-cause death.

Rates of all-cause mortality within 8-day and 30-day post-MI were lower in the RT patients than in non-RT patients (6.7% vs. 11.4%, *p* < 0.0001 and 11.1% vs. 18.0%, *p* < 0.0001, respectively) (Table 2). Non-adjusted cumulative incidence of all-cause death during the whole follow-up was lower in RT patients compared to non-RT patients (HR 0.86, 95% CI 0.77–0.95 for RT patients). However, after multivariable analysis, RT was independently associated with a higher risk for all-cause death (adjusted hazard ratio 1.15, 95% CI 1.03–1.28) (Figure 4).

When considering cardiovascular mortality during the eight days, 30 days post MI, the RT patients also had a lower unadjusted rate of CV mortality (5.3% vs. 8.5%, *p* = 0.0002; 7.8%, vs. 12.3%, *p* < 0.0001 respectively). (Table 2). Non-adjusted cumulative incidence of cardiovascular death during the whole follow-up was also lower in RT patients compared to non-RT patients (HR 0.79, 95% CI 0.68–0.92 for RT). After multivariable analysis, RT was associated with a non-significant higher risk for cardiovascular death (adjusted hazard ratio 1.12, 95% CI 0.95–1.32). Fine and Gray model for competing risks of cardiovascular and non-cardiovascular death provided similar findings (hazard ratio 1.07, 95% CI 0.91–1.26).

## 4. Discussion

Our study, identifying 94,340 MI patients over a 5-year follow up period from a baseline population of 3,306,383 patients, showed relevant findings: a 45% higher risk of acute myocardial infarction in RT patients after multivariate adjustment; specific profiles of MI with kidney transplantation; and a 15% higher risk for all-cause mortality among RT patients after MI.

To the best of our knowledge, this was the first study carried out among such a nationwide population of RT from all hospitals performing kidney transplantations (after excluding patients on dialysis without kidney transplant). The first large cohort, published in 2004 included 60,141 RT patients and 66,813 adult kidney wait listed patients, and showed two major elements: high CV mortality in the first three months post-transplantation, and the impact of the pre-transplantation duration of dialysis on cardiovascular mortality [13]. The authors also underlined that CV mortality fell after the third month post-transplantation and reached a level below that of patients still on the waiting list [13]. Kasiske et al. in 2006, also reported an excess risk of MI in the first month post-transplantation. Nonetheless, this study did not provide long-term prognosis of MI in RT patients. [7].

During the whole follow-up period after MI, non-adjusted analysis reported a lower risk of all-cause mortality in RT patients than in non-RT patients (who were more than 10 years older). Nevertheless, adjusted cumulative incidence of all-cause of death showed a 15% higher risk for all-cause mortality among RT patients after MI (Figure 4).

The trend of 5.8% MI in RT patients at around five years found in our study is consistent with the literature. In a recent multicenter study conducted in Canada, incidence of MI in RT patients was 4% after 4.5 years of follow-up. Trends were significantly higher than in non-RT patients, and these results were consistent after adjustment [14].

In our study, median time from kidney transplantation to the onset of the MI was 70 months (IQR 48–90 month, Figure 4), meaning that 25% of MI in RT patients occurred in the first four years post transplantation. This slightly differs from the literature, but considers the risk of cardiovascular events during the first years post-transplantation. The structure of our analysis may also explain this apparent difference since we evaluated the incidence of MI with a relatively long follow-up in contrast to studies about the prevalence of MI with the history of RT identified in a shorter period before the index event.

Humar et al. described a risk of peri-operative cardiac events of 6%, and notably a risk of MI of 1.6% among the 2694 kidney transplantations at the University of Minnesota over 14 years [15]. Claes et al. reported an incidence of 3.3% of cardiac events in the short-term in a study of 331 RT patients at the University Hospitals of Leuven [16]. Finally, Lentine reviewed 35,847 patients using the United States Renal Data System (USRDS) and found that the post-operative incidence of MI (using administrative records) was 4.3% [10].

Patients with RT were more frequently admitted with MI, and non-ST elevation myocardial infarction (NSTEMI) was more frequent than in patients with no RT whereas ST elevation myocardial infarction (STEMI) incidence was conversely lower. This was also reported in the study of Agrawal et al. [17].

Patients with RT were significantly younger at the time of the MI. A combination of pathophysiologic and clinical data may explain the younger onset of MI in RT patients:

- Their high CV risk profile, with higher prevalence of CV risk factors (diabetes, hypertension, dyslipidemia).
- The prevalence of vascular disease was higher in RT patients compared to non-RT patients admitted with MI. This finding confirms that patients requiring kidney transplantation have a high prevalence of diffused atherosclerotic disease, suggesting that kidney disease is globally associated with an increased risk of atherosclerosis. This association is in keeping with the results of the study by Gill et al., who showed that the presence of peripheral arterial disease was associated with significantly increased cardiovascular risk [9].
- The higher prevalence of a history of coronary artery disease in kidney transplant patients, which we also found in our study, suggests a more rapid progression of the atherosclerotic process, probably related to chronic inflammation, whose consequences may notably include an increased CV risk [18,19].
- The immediate post-operative period is associated with significant hemodynamic stress in a pro-inflammatory milieu, which can promote the destabilization of atheromatous plaque and endothelial dysfunction, thus leading to MI [20]. This period is associated with an exacerbation of factors that contributes to increased shear stress in coronary arteries, leading not only to plaque rupture and thrombus formation, but also to an increase in myocardial demand for oxygen. There is also a state of acquired thrombophilia after transplantation. The use of immunosuppressive drugs, viral infections, and the interruption of anticoagulants in the first weeks following the transplantation may also play a role in the pro-thrombotic state [15,16,20].
- Moreover, anemia was more frequently observed in the RT patients whilst the role of anemia in type 2 NSTEMI has clearly been established [21].
- Finally, Kasiske showed that MI are more frequent when kidneys come from dead donors than alive donors [7]. Unfortunately, this information is not available in the PMSI database and could therefore not be included in our analysis.

These clinical characteristics appear to be major criteria that may help risk stratification in these patients, and support the use of models like the PORT model to evaluate the risk of CV events in the year following transplantation [12]. Traditional scores, like the Framingham score, have some limitations among patients with kidney failure and those with kidney transplants. CV risk in these patients is most often underestimated, thereby requiring the use of several specific scores that currently only assess the risk of major cardiovascular events (MACE) in the long-term. Risk evaluations should therefore give greater weight to cardiovascular history, hypertension, and diabetes, as with the INDANA Score [22].

The prevalence of traditional cardiovascular risk factors was very high in our series, which is in agreement with the literature [4,5,6,7]. Indeed, the majority were men (69.4%) and the percentages of diabetes and hypertension were high, which are again consistent with the literature [22,23,24,25,26].

The impact of some treatments used after renal transplantation on blood pressure, glycemia, or factors of thrombosis may also play a role. Patients treated with anti-calcineurins have a major impact on blood pressure. In addition, patients treated with steroids or mTor inhibitors often have marked lipid disorders [26,27,28,29].

### 4.1. Impact of Screening or Treatments

Prevention of post-transplantation CV events often depends on perioperative management. Prior to transplantation, a CV evaluation is highly recommended by validated guidelines including clinical evaluation, electrocardiogram, and cardiac echocardiography. Further explorations may be needed including invasive and non-invasive testing in the presence of more than three risk factors (diabetes, hypertension, dyslipidemia, smoking, prior CV disease, one year or more on dialysis, left ventricular hypertrophy, and age above 60 years) or in patients with clinical symptoms of coronary ischemia [30].

Ohtake et al. studied 30 patients at the moment hemodialysis was initiated. All underwent coronary angiography, and >50% had intermediate coronary stenosis while 83% of the diabetic patients had significant ones [31]. In another short series of 63 patients, these results were confirmed with 49% of patients (including asymptomatic ones) having significant coronary artery disease. The author showed a relationship between coronary lesions and the cardiovascular events over a 5-year follow-up [32]. Screening for ischemia and treating it are thus major objectives before renal transplantation. Finally, a large series showed that a dobutamine stress echocardiography revealed ischemia in a third of cases. It was associated with a significantly higher risk of MACE at one year, despite revascularization, with 22.4% vs. 3.4% (*p* < 0.001) of MI and 19.4% vs. 4.8% (*p* < 0.001) of ventricular arrhythmias in patients with a positive and negative stress test, respectively [33]. However, in a recent study, abnormal scintigraphy was associated with more revascularization of ischemic lesions than abnormalities shown with other diagnostic tests. Revascularization was required in 21/60 (35.0%) of patients undergoing coronary angiography, and revascularization indication increased by 50% when coronary angiography was performed because of abnormal scintigraphy [34]. Moreover, in a different study published in 2018, pre-renal transplantation abnormal myocardial perfusion was an independent predictor of post-transplantation CV events [33]. The time between the screening for myocardial ischemia and the transplantation as well as the frequency of screening is a matter of debate. Indeed, the preoperative examination is often performed before the patient is added to the transplant waiting list and patients remain on the waiting list for years, exposing them to a greater degree of oxidative stress, which may explain the higher prevalence of ischemic heart disease at the time of the transplantation.

This study recalls the importance of the screening of cardiovascular risks during the pre-transplantation and the transplantation period because it can lead to primary prevention treatments.

### 4.2. Study Limitation

This study has some limitations. Comorbidities of patients were recorded at discharge from hospital in this nationwide database. Variables may have been under-coded, but this process probably affected both groups to the same degree. It must be pointed out that the prospective payment system encourages hospitals to collect diagnoses and associated procedures exhaustively. The use of the database for the funding of French healthcare facilities has improved over the years, resulting in better coding. Unfortunately, little quantitative information is available and we were unable to identify patients treated with immunosuppressive drugs. All immunosuppressive drugs increased CV risk by potentiating traditional risk factors. The left ventricular ejection fraction and details for coronary artery lesions were not recorded, even though they are known to be prognostic factors in these patients.

## 5. Conclusions

MI is not an uncommon complication after RT (incidence of around 5.8% after 5 years). RT is independently associated with a 45% higher risk of MI than in non-RT patients, with a predominance of NSTEMI. In the case of MI, RT is independently associated with a 15% higher risk of all-cause death.

## Figures and Tables

**Table 1:** Baseline characteristics of patients admitted in French hospitals in 2013 with at least a 5-year follow-up.

	Total (n = 3,306,383)	No RT (n = 3,288,857)	RT (n = 17,526)	*p*
Age (years), (m ± SD)	59.1 ± 21.6	59.1 ± 21.6	55.4 ± 14.5	<0.0001
Gender (male)	1,538,428 (46.5)	1,527,851 (46.5)	10,577 (60.4)	<0.0001
Hypertension	981,355 (29.7)	969,014 (29.5)	12,341 (70.4)	<0.0001
Diabetes mellitus	437,463 (13.2)	432,503 (13.2)	4960 (28.3)	<0.0001
Smoker	224,978 (6.8)	223,715 (6.8)	1263 (7.2)	0.03
Dyslipidemia	423,169 (12.8)	418,773 (12.7)	4396 (25.1)	<0.0001
Obesity	342,223 (10.4)	340,066 (10.3)	2157 (12.3)	<0.0001
Heart failure	331,104 (10.0)	327,218 (9.9)	3886 (22.2)	<0.0001
History of pulmonary edema	23,180 (0.7)	22,504 (0.7)	676 (3.9)	<0.0001
Valvular heart disease	115,792 (3.5)	114,746 (3.5)	1046 (6.0)	<0.0001
Previous endocarditis	4095 (0.1)	4011 (0.1)	84 (0.5)	<0.0001
Dilated cardiomyopathy	72,322 (2.2)	71,411 (2.2)	911 (5.2)	<0.0001
Coronary artery disease	340,834 (10.3)	337,422 (10.3)	3412 (19.5)	<0.0001
Previous myocardial infarction	54,777 (1.7)	54,371 (1.7)	406 (2.3)	<0.0001
Previous PCI	84,560 (2.6)	83,883 (2.6)	677 (3.9)	<0.0001
Previous CABG	11,426 (0.3)	11,267 (0.3)	159 (0.9)	<0.0001
Vascular disease	272,496 (8.2)	269,344 (8.2)	3152 (18.0)	<0.0001
Atrial fibrillation	311,035 (9.4)	309,040 (9.4)	1995 (11.4)	<0.0001
Ischemic stroke	61,970 (1.9)	61,688 (1.9)	282 (1.6)	0.01
Alcohol related diagnoses	183,092 (5.5)	182,587 (5.6)	505 (2.9)	<0.0001
Chronic kidney disease	72,510 (2.2)	60,164 (1.8)	12,346 (70.4)	<0.0001
Lung disease	331,899 (10.0)	330,417 (10.0)	1482 (8.5)	<0.0001
Sleep apnea syndrome	128,674 (3.9)	127,994 (3.9)	680 (3.9)	0.94
COPD	180,632 (5.5)	179,948 (5.5)	684 (3.9)	<0.0001
Inflammatory disease	171,715 (5.2)	169,675 (5.2)	2040 (11.6)	<0.0001
Anemia	258,380 (7.8)	251,824 (7.7)	6556 (37.4)	<0.0001
Death during follow-up	916,966 (27.7)	912,462 (27.7)	4504 (25.7)	<0.0001
Cardiovascular death	184,216 (5.6)	183,157 (5.6)	1059 (6.0)	0.01

Data are expressed as n (%) or m ± SD. RT: renal transplant. PCI: percutaneous coronary intervention. CABG = coronary artery bypass graft. COPD: chronic obstructive pulmonary disease.

**Table 2:** Characteristics of patients admitted for acute myocardial infarction.

	Total (n = 94,340)	No RT (n = 93,320)	RT (n = 1020)	*p*
Age, years	70.5 ± 13.3	70.6 ± 13.3	59.5 ± 11.5	<0.0001
Gender (male)	59,269 (62.8)	58,561 (62.8)	708 (69.4)	<0.0001
Hypertension	45,679 (48.4)	44,904 (48.1)	775 (76.0)	<0.0001
Diabetes mellitus	23,955 (25.4)	23,560 (25.2)	395 (38.7)	<0.0001
Smoker	8619 (9.1)	8522 (9.1)	97 (9.5)	0.68
Dyslipidemia	21,977 (23.3)	21,638 (23.2)	339 (33.2)	<0.0001
Obesity	12,130 (12.9)	11,979 (12.8)	151 (14.8)	0.06
Heart failure	16,679 (17.7)	16,350 (17.5)	329 (32.3)	<0.0001
History of pulmonary edema	636 (0.7)	592 (0.6)	44 (4.3)	<0.0001
Valvular heart disease	5309 (5.6)	5232 (5.6)	77 (7.5)	0.01
Previous endocarditis	151 (0.2)	142 (0.2)	9 (0.9)	<0.0001
Dilated cardiomyopathy	3086 (3.3)	3023 (3.2)	63 (6.2)	<0.0001
Coronary artery disease	23,752 (25.2)	23,390 (25.1)	362 (35.5)	<0.0001
Previous PCI	6032 (6.4)	5952 (6.4)	80 (7.8)	0.06
Previous CABG	575 (0.6)	562 (0.6)	13 (1.3)	0.01
Vascular disease	16,421 (17.4)	16,103 (17.3)	318 (31.2)	<0.0001
Atrial fibrillation	11,522 (12.2)	11,383 (12.2)	139 (13.6)	0.17
Ischemic stroke	2582 (2.7)	2559 (2.7)	23 (2.3)	0.34
Alcohol related diagnoses	5210 (5.5)	5182 (5.6)	28 (2.7)	0.0001
Chronic kidney disease	3619 (3.8)	2865 (3.1)	754 (73.9)	<0.0001
Lung disease	12,171 (12.9)	12,099 (13.0)	72 (7.1)	<0.0001
Sleep apnea syndrome	5133 (5.4)	5069 (5.4)	64 (6.3)	0.24
COPD	7932 (8.4)	7893 (8.5)	39 (3.8)	<0.0001
Inflammatory disease	5412 (5.7)	5254 (5.6)	158 (15.5)	<0.0001
Anemia	7979 (8.5)	7599 (8.1)	380 (37.3)	<0.0001
STEMI	46,283 (49.1)	45,835 (49.1)	448 (43.9)	0.001
NSTEMI	48,057 (50.9)	47,485 (50.9)	572 (56.1)	0.001
Anterior MI	23,738 (25.2)	23,523 (25.2)	215 (21.1)	0.003
Inferior MI	15,454 (16.4)	15,302 (16.4)	152 (14.9)	0.2
MI with other location	55,148 (58.5)	54,495 (58.4)	653 (64.0)	0.0003
HF at the acute phase	22,380 (23.7)	22,200 (23.8)	180 (17.6)	<0.0001
Pulm. edema/shock at the acute phase	7808 (8.3)	7724 (8.3)	84 (8.2)	0.96
PCI during first 8 days	41,619 (44.1)	41,189 (44.1)	430 (42.2)	0.21
Death day 0–8 post MI	10,685 (11.3)	10,617 (11.4)	68 (6.7)	<0.0001
Death day 0–30 post MI	16,864 (17.9)	16,751 (18.0)	113 (11.1)	<0.0001
Cardiovascular death day 0–8 post MI	8012 (8.5)	7958 (8.5)	54 (5.3)	0.0002
Cardiovascular death day 0–30 post MI	11,532 (12.2)	11,452 (12.3)	80 (7.8)	<0.0001

Data are expressed as n (%) or m ± SD. RT: renal transplant. PCI: percutaneous coronary intervention. CABG: coronary artery bypass graft. COPD: chronic obstructive pulmonary disease. MI: myocardial infarct, STEMI: ST elevation myocardial infarct. NSTEMI: Non ST elevation myocardial infarct.
